# System in biology leading to cell pathology: stable protein-protein interactions after covalent modifications by small molecules or in transgenic cells

**DOI:** 10.1186/1423-0127-18-7

**Published:** 2011-01-19

**Authors:** Halina Z Malina

**Affiliations:** 1 MalinaLab-Axanton, Tiefenaustr.110, CH-3004 Bern, Switzerland

## Abstract

**Background:**

The physiological processes in the cell are regulated by reversible, electrostatic protein-protein interactions. Apoptosis is such a regulated process, which is critically important in tissue homeostasis and development and leads to complete disintegration of the cell. Pathological apoptosis, a process similar to apoptosis, is associated with aging and infection. The current study shows that pathological apoptosis is a process caused by the covalent interactions between the signaling proteins, and a characteristic of this pathological network is the covalent binding of calmodulin to regulatory sequences.

**Results:**

Small molecules able to bind covalently to the amino group of lysine, histidine, arginine, or glutamine modify the regulatory sequences of the proteins. The present study analyzed the interaction of calmodulin with the BH3 sequence of Bax, and the calmodulin-binding sequence of myristoylated alanine-rich C-kinase substrate in the presence of xanthurenic acid in primary retinal epithelium cell cultures and murine epithelial fibroblast cell lines transformed with SV40 (wild type [WT], Bid knockout [Bid-/-], and Bax-/-/Bak-/- double knockout [DKO]). Cell death was observed to be associated with the covalent binding of calmodulin, in parallel, to the regulatory sequences of proteins. Xanthurenic acid is known to activate caspase-3 in primary cell cultures, and the results showed that this activation is also observed in WT and Bid-/- cells, but not in DKO cells. However, DKO cells were not protected against death, but high rates of cell death occurred by detachment.

**Conclusions:**

The results showed that small molecules modify the basic amino acids in the regulatory sequences of proteins leading to covalent interactions between the modified sequences (e.g., calmodulin to calmodulin-binding sites). The formation of these polymers (aggregates) leads to an unregulated and, consequently, pathological protein network. The results suggest a mechanism for the involvement of small molecules in disease development. In the knockout cells, incorrect interactions between proteins were observed without the protein modification by small molecules, indicating the abnormality of the protein network in the transgenic system. The irreversible protein-protein interactions lead to protein aggregation and cell degeneration, which are observed in all aging-associated diseases.

## Background

Cell degeneration is observed in all aging- and infection-associated pathologies. Currently, the same process of apoptosis is considered to occur in tissue homeostasis and development, as well as in diseases. The current understanding suggests that too little apoptosis leads to cancer and too much apoptosis leads to degenerative diseases. Consequently, cancers are treated with small molecules to induce apoptosis; however, prolonged use of small molecules also leads to cancer [1].

This understanding of apoptosis in disease development did not give a solution for the treatment of degenerative diseases and led to very toxic methods in cancer therapeutics. The understanding of apoptosis is a key issue for further research.

Many attempts have been made to heal aging-associated diseases by inhibiting the caspases. The failure of this approach indicates that cell degeneration cannot be stopped by inhibition of the end-enzyme caspase. Therefore, an upstream event is responsible for cell degenerative disorders.

Knowledge of the mechanism associated with pathological apoptosis is necessary to stop aging-associated degeneration, which is a feature of aging-associated pathologies. The lesson from cancer showed that treatment with small molecules leads to degenerative diseases in other organs. The cells do not disappear, but protein aggregates are formed leading to complications in the therapy, such as thrombosis and kidney degeneration [2-5].

Aging-associated degeneration is accelerated with pollution. Reactive oxygen species (ROS) caused by pollution have been reported as a major factor for degenerative diseases [6]. Oxidative stress has been considered for years as a cause of diseases. Oxidative stress leads to induction of indoleamine-2,3-dioxygenase and production of kynurenines and the end product xanthurenic acid [7]. The fluorescence of the lens proteins has been used for cataract diagnosis since the 19th century. Thus, small molecules are a very important factor leading to degenerative diseases. Degenerative diseases are associated with aging, indicating accumulation of the changes caused by small molecules.

The current study showed that small molecules, such as xanthurenic acid modifying the regulatory sequences of proteins, lead to stable interactions between proteins and a new pathological network, which we called misfoldome.

Xanthurenic acid, an endogenous substance formed from tryptophan, is the small molecule in this cell culture model of disease development by posttranscriptional modification of the proteins in neuromodulation [8], but its covalent binding with proteins leads to cell death [9]. Exposure to xanthurenic acid at concentrations of 10 μM or higher for more than 72 hours has been observed to lead to pathological apoptosis [10] and oxidative stress [11,12]. It has previously been reported that xanthurenic acid accumulates in senile cataract [13], leading to an unfolded protein response [9]. Normal physiological apoptosis is a regulated process based on a network of reversible interactions between proteins, called interactome. The basic requirement for this protein network is the regulated interactions between proteins. The flexible interactions maintain cell physiology.

Xanthurenic acid forms an oxidative derivative, an amino-quinone [14,15], and the quinone radicals react with the amine group of proteins. This chemical reaction could be responsible for the modification of proteins in a time- and concentration-dependent manner, leading to aging-associated diseases. The misfolded proteins change their place and role in the cells, leading to irreversible pathological apoptosis, mitochondrial damage, interruption of calcium homeostasis, and translocation of the signaling protein 14-3-3 into lysosomes [10-12,16]. Pathological apoptosis is induced through the mitochondrial pathway, which involves translocation of the BH3-only proapoptotic proteins into the mitochondrial membrane, leading to caspase-3 activation. The interaction of 14-3-3 with phosphorylated Bad is interrupted, leading to Bad dephosphorylation and translocation into mitochondria. These events were also described for apoptosis; however, in pathological apoptosis, they are not regulated, leading to a constitutive degenerative process.

The present study shows that the covalent modifications of proteins by xanthurenic acid lead to covalent, and subsequently nonregulated, interactions of calmodulin with the binding sites regulated by calmodulin and/or phosphatidylinositol-4,5-phosphate, such as the effector domain (ED) sequence of myristoylated alanine-rich C-kinase substrate (MARCKS) and BH3 of Bax. The covalent interactions between signaling proteins, such as calmodulin and the calmodulin-binding sites of the proteins, abolish tissue homeostasis. The new, stable signaling of the network of calmodulin-binding proteins, which regulate hundreds of proteins, leads to many pathological events in parallel, and this is observed in all degenerative diseases.

## Methods

### Reagent

*N*-tert-Butyloxycarbonyl-amino acids (Boc-amino acids), xanthurenic acid, and other chemicals were purchased from Sigma (Buchs, Switzerland), and the peptides were synthesized by Virusys (USA). The rabbit polyclonal antibodies against the peptides, modified *in vitro*, were prepared at the University of Zurich (Switzerland). Antibodies against PARP, caspase-3, the N-terminal part of Bax, calmodulin, and rabbit secondary IgG were from Santa Cruz Biotechnology Inc. (Santa Barbara, CA, USA); phospho-Bad Ser136 was from Cell Signaling Technology Inc. (Danvers, MA, USA). Mitotracker CMXRos was from Molecular Probes (Leiden, The Netherlands).

### Cell culture

Primary cell cultures of human retinal epithelial cells (RPE) were prepared and cultivated as previously described [11]. Murine embryonic fibroblast (MEF) cell lines, wild type (WT), Bid knockout (Bid-/-), and Bax-/-/Bak-/- double knockout (DKO), were provided by Dr. S. J. Korsmeyer (Harvard Medical School, USA). The cell cultures were cultivated in MEM from Invitrogen (Basel, Switzerland).

### Immunoprecipitation

The cell extracts were mixed with the antibody at a concentration of 10 μg/mL overnight at 4°C, and the immunoprecipitated proteins were separated on SDS-PAGE gel. The proteins were detected by western blot analysis with the appropriate antibodies.

### Western blot analysis

Control cells and cells from the cell culture in the presence of increasing concentrations of xanthurenic acid (5, 10, and 20 μM of medium) were allowed to grow in parallel in a 750-mL flask. The cells were washed in PBS and lysed in 50 mM Tris-Cl (pH 8), 150 mM NaCl, 1% Triton X-100, and the following protease inhibitors: 0.1 mM phenyl-methylsulfonyl fluoride and 1 μg/mL each of leupeptin, pepstatin and aprotinin. Then, the sample was centrifuged for 10 min at 14,000*g*, and the supernatant was boiled in loading buffer for 5 min. Proteins at 100 μg per lane were separated by SDS-PAGE containing 12.5% acrylamide. The proteins, after being transferred to Hybond, were probed with the appropriate antibodies. The chemiluminescence ECL system (Amersham Pharmacia Biotech AB, Uppsala, Sweden) was used for the detection of peroxidase-conjugated secondary antibody. Xanthurenic acid induced pathological apoptosis, leading to degradation of the cell proteins with parallel aggregation of the basic protein sequences. The same global quantities of proteins in the cell extracts were compared. Loading with proteins known to be degraded during apoptosis, such as actin, will lead to interpretation errors.

### Immunofluorescence studies

Cells grown on glass coverslips were fixed for 10 min at room temperature in 4% paraformaldehyde in 0.1 M PIPES (pH 6.8), washed in PBS, and permeabilized for 5 min in PIPES containing 0.05% saponin (65 μL per coverslip). The cells were then washed in PBS, incubated for 10 min in cold acetone for additional fixing and permeabilization, and again washed in PBS. The cells were incubated for 1.5 h with the first antibody diluted in PBS containing 1% bovine serum albumin, and after further washing, the cells were incubated for 1.5 h with the secondary antibody. The coverslips were washed in PBS and incubated for 10 min with 65 μL of solution containing 1 mL of Hoechst 33342 dye (1 mg/mL), washed in PBS, and incubated with Antifade Kit (Molecular Probes, Leiden, the Netherlands) according to the supplier's instructions. Mitotracker was used as previously described [11].

### Microscopy

Confocal microscopy was done with a Zeiss 410 laser scanning microscope (Department of Clinical Research, University of Bern, Switzerland).

## Results

### Xanthurenic acid modifies the Boc-amino acids lysine, arginine, histidine, and glutamine

The Boc modification of amino acids blocks its primary amino group, and the secondary amino group of amino acids remains free from modification. The Boc-amino acids lysine, arginine, histidine, and glutamine were observed to bind covalently with fluorescent xanthurenic acid. The covalently modified, fluorescent amino acids were separated by thin layer chromatography. The result showed that a reaction between xanthurenic acid and Boc-amino acid occurs on the secondary groups of amino acids and is dependent on xanthurenic acid concentration and incubation time. Consequently, the amino groups in proteins and peptides are modified by xanthurenic acid, leading to their polymerization. Covalent cross-linking of polylysine, for example, was observed on SDS-PAGE gels after incubation with xanthurenic acid. The chemical reaction of the covalent modification of the proteins by xanthurenic acid occurs in cell culture *in vitro*, leading to fluorescent proteins. Xanthurenic acid is an endogenous substance, and the modification of the proteins *in vivo *leads to accumulation of the modified, fluorescent proteins, as observed in pathological tissues such as the cataract lens. The proteins modified covalently by small molecules on the secondary amino group of amino acids became stably misfolded and insoluble. The modification occurs in random on 1 or several amino acids, making the establishment of the structure of the modified protein difficult or impossible. However, knowledge of the protein structure after modification, which could be of interest to chemists, has less importance for cell biologists and drug development researchers. In this study, the relevant fact is that the modification leads to stable protein-protein interactions inducing nonregulated signaling. The network, which cannot be further regulated, leads to cell pathology.

### Cell death in the presence of xanthurenic acid in MEF cell lines

**Figure 1:**
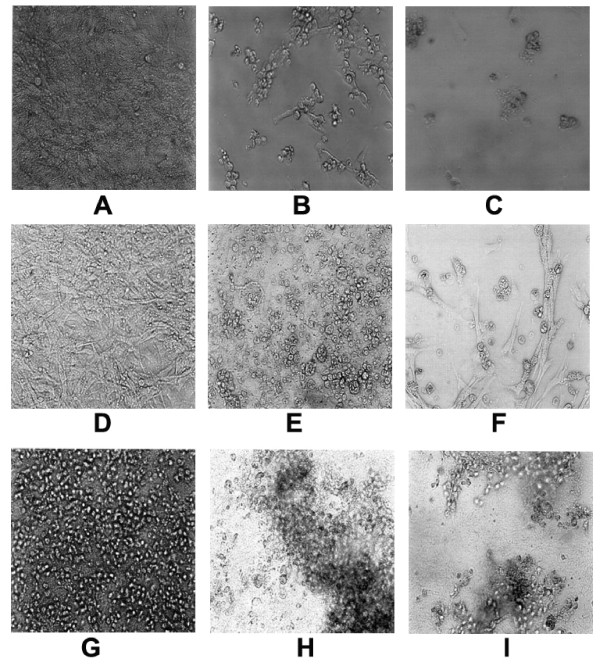
**MEF cell lines death, WT, Bid -/- and DKO, in the presence of xanthurenic acid (Xan)**: (A, B, C)- wide type; (D, E, F) -Bid-/-; (G, H, I)- DKO. (A, D, G)- control; (B, E, H)-10 μM Xan; (C, F, I) -20 μM Xan.

**Figure 2:**
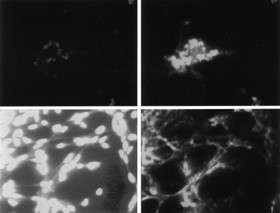
**MEF cell line, BID -/-: activation of PARP and caspase 3 in the presence of 10 μM of xanthurenic acid**: upper photos the cells without xanthurenic acid and bottom photos with 10 μM of xanthurenic acid: left (upper-down) detection of PARP, right (upper-down) detection of caspase-3.

Xanthurenic acid induced apoptotic cell death in MEF cells (Figure 1). Cell death was caspase-3 and PARP dependent in WT MEF cells. Caspase-3 and PARP were not activated by xanthurenic acid in DKO cells, but in Bid-/- cells, an activation of PARP and caspase-3 was observed (Figure 2A,B). Thus, Bax and Bak are essential for the induction of caspase-3 in the presence of xanthurenic acid in cell lines transformed with SV40. However, the lack of proapoptotic proteins in the Bax/Bak double knockout did not rescue the cells from death in the presence of xanthurenic acid; cell death occurred by massive detachment. This indicates that xanthurenic acid leads to activation of an upstream target common for caspase-3 activation and cell detachment.

### Covalent protein-protein interactions in the xanthurenic acid cell culture model

It was of interest to determine the covalent protein-protein interactions leading to pathological apoptosis and to establish the conditions necessary for the covalent modification of the proteins in the cell leading to protein polymerization. The xanthurenic acid in the primary cell culture was used as a model of cell pathology development in the presence of small molecules. We studied the mechanism of pathological apoptosis in the presence of xanthurenic acid in the RPE primary cell culture and the cell culture of MEF cell lines transformed with SV40. In cell culture with 10 μM xanthurenic acid, the covalent modification of the proteins began after 72 h. In the same conditions, the covalent interactions, clearly detectable by western blot, were observed after 96-120 h. The 120-hour cell culture was then used, in which the covalent interactions were easily detectable.

### Absence of Bad protein dephosphorylation in MEF cell lines transformed with SV40 in the presence of xanthurenic acid

**Figure 3:**
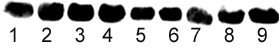
**Western blot analysis of pBad immunoprecipitated with 14-3-3 in MEF cells: WT, Bid -/- and DKO**: WT lanes 1-3; 1-control, lanes (2-3) in the presence of xanthurenic acid: lane 2-10 μM, lane 3- 20 μM; Bid -/-: lane 4-control, lanes (5-6) in the presence of xanthurenic acid: lane 5-10 μM, lane 6- 20 μM; DKO lanes 7-9; lane 7-control, lanes (8-9) in the presence of xanthurenic acid: lane 8-10 μM, lane 9- 20 μM.

Previously, it has been reported that Bad is dephosphorylated with 10 μM xanthurenic acid and translocated into mitochondria in primary astrocytes in the presence of xanthurenic acid [16]. In this study, Bad was analyzed in MEF cell cultures (WT, Bid-/-, and DKO). Bad interaction with 14-3-3 proteins prevents apoptosis, and phosphorylation of Bad Ser136 is crucial for its binding to 14-3-3 [17]. Western blot analysis of the WT, Bid-/-, and DKO protein extracts from control cells and cells growing in the presence of 10 and 20 μM xanthurenic acid showed that phospho-Bad Ser136 was not dephosphorylated in these cell lines in the presence of xanthurenic acid (Figure 3). This indicates that Bad does not play a role in the induction of apoptosis in these cells transformed with SV40 (Figure 3). The study also indicates that the results obtained in a cell line are different from those obtained in a primary cell culture, which mimics the conditions in the normal mammalian tissue.

### Covalent interaction of MARCKS with calmodulin in Bid-/- and DKO MEF cells

**Figure 4:**
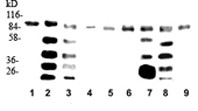
**Interaction of calmodulin-binding site of MARCKS with calmodulin (CAM) in the presence of xanthurenic acid in MEF cells WT, Bid -/- and DKO.** Western blot analysis with antibody against ED sequence of MARCKS of proteins immunoprecipitated with CAM: WT lanes1-3, lane 1-control, lanes (2-3) in the presence of xanthurenic acid: lane 2-10 μM, lane 3- 20 μM; Bid -/- lanes 4-6: lane 4-control, lanes (5-6) in the presence of xanthurenic acid: lane 5-10 μM, lane 6- 20 μM; DKO lanes 7-9; lane 7-control, lanes (8-9) in the presence of xanthurenic acid: lane 8-10 μM, lane 9- 20 μM.

The proteins were immunoprecipitated from extracts of MEF cell lines (WT, Bid-/-, and DKO) with an antibody against calmodulin (Figure 4) Western blot analysis was used to investigate the binding of calmodulin to MARCKS proteins. Calmodulin interactions with the basic sequence of the ED of MARCKS became covalent in the presence of xanthurenic acid, but they were different in the knockout cells in comparison to WT cells. The ED of MARCKS is an example of a lysine-rich regulatory sequence. The ED binds calmodulin electrostatically. MARCKS with bound calmodulin is translocated to acidic membranes and cannot be phosphorylated by protein kinase C [18]. Calmodulin trapped by MARCKS cannot participate in the numerous calmodulin functions [18].

We now report that in the presence of xanthurenic acid, calmodulin binds covalently to the ED sequence of MARCKS (Figure 4). Wild-type, Bid-/-, and DKO MEF cells were used. The interaction between calmodulin and the ED sequence of MARCKS in these cells was investigated. Cell extracts from the MEF cells, WT, Bid-/-, and DKO from control cells and cells grown with 10 and 20 μM xanthurenic acid, were immunoprecipitated with calmodulin. The immunoprecipitated proteins were analyzed by western blot with antibody against the ED domain of MARCKS (KKKKKRFSFKKSFKLSGFSFKKKNKK). Calmodulin did not bind covalently MARCKS in control cells. However, in the presence of 10 and 20 μM xanthurenic acid, covalent crosslinking of calmodulin with MARCKS occurred.

In the presence of xanthurenic acid, the small protein calmodulin (18.6 kDa) binds covalently to the ED sequence of the degraded MARCKS and forms numerous polymers. The results showed that the protein aggregation takes place despite cell degeneration (Figure 1). Calmodulin forms stable polymers with the calmodulin-binding sites of MARCKS, which are not degraded by cell death-associated proteases but form aggregates. The western blot showed that the polymers were better detectable in the presence of 10 μM compared with 20 μM xanthurenic acid. This result suggests that with a higher concentration of xanthurenic acid, the polymers formed aggregates with other proteins, becoming partly insoluble and not present in the cell-free extract and/or less accessible for binding with calmodulin.

In the same cells, the interaction of MARCKS with calmodulin was completely different between the Bid-/- and DKO genotypes. In double-mutant cells (DKO), MARCKS bound calmodulin in control cells and in the presence of 10 μM xanthurenic acid, but this interaction was abolished at 20 μM xanthurenic acid. In Bid-/- cells, the MARCKS-calmodulin covalent interaction was not detected.

The results indicate that xanthurenic acid abolishes the regulation of cell physiology by generating covalent interactions between proteins. The protein-protein interactions in knockout cells are different from those in WT cells. The results suggest that it would be inappropriate to use knockout animal models to study the development of aging-associated disease processes or drugs against metabolic diseases, such as atherosclerosis, Alzheimer's disease, Parkinson's disease, retinal degeneration etc., because the protein-protein interactions can be different from those in aging mammalian having the posttranscriptionally modified proteins.

### Xanthurenic acid interrupts BH3 interaction with 14-3-3 and causes new covalent Bax-calmodulin interactions in the primary cell culture of RPE translocated to the mitochondrial membrane

**Figure 5:**
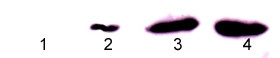
**Binding of calmodulin (CAM) to BH3 sequence of Bax in the presence of xanthurenic acid in the primary human cell culture of the human retinal epithelial cells**: Western blot analysis with antibody against BH3 of proteins immunoprecipitated with CAM, lane 1-control, lanes (2-4) in the presence of xanthurenic acid: lane 2-5 μM, lane 3-10 μM, lane 4- 20 μM.

It was previously reported that Bax in the cytoplasm binds 14-3-3-θ protein, and an interruption of this noncovalent binding leads to Bax translocation into mitochondria [19]. An antibody was prepared against the BH3 sequence -KKLSECLKR- of Bax polymerized with xanthurenic acid, but without conjugation with another protein, then specific only for the BH3 sequence of Bax. Anti-calmodulin antibody was used for the precipitation of protein extracts from retinal pigment epithelium primary cell cultures and cultures grown in the presence of xanthurenic acid. Western blot analysis of the immunoprecipitated proteins showed a band of 37 kDa, which reacts with the anti-BH3 sequence of Bax. This indicates that Bax and calmodulin are covalently bound in the presence of xanthurenic acid (Figure 5).

**Figure 6:**
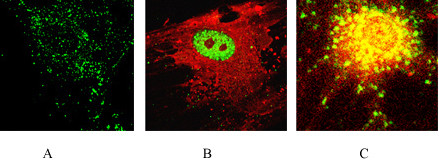
**Primary cell culture of the human retinal epithelial cells: localization of BH3 sequence in the presence of xanthurenic acid**: (A) BH3 sequence (green) in the presence of 10 μM xanthurenic acid, (B) NH2-terminal sequence (green) in the presence of 20 μM xanthurenic acid is translocated to nucleus, Mitotracker (red) shown polymerization of mitochondrial membranes, (C) mitochondria (yellow): co-localization of BH3-sequence (green) in presence of 20 μM xanthurenic acid and with Mitotracker (red).

Confocal microscopy showed that the BH3 sequence in the presence of 10 μM xanthurenic acid was detected in mitochondria (Figure 6A); at 20 μM xanthurenic acid, the BH3 sequence was not further translocated into the nucleus but colocalized in the mitochondrial membrane (Figure 6B). Bax, in the absence of xanthurenic acid was in cytoplasm, as detected with the same antibody anti-N terminal sequence of Bax (Santa Cruz) and reported by Malina et al. [12]. However, in the presence of 20 μM xanthurenic acid the N-terminal part of Bax colocalized in the nucleus (Figure 6C). It suggests stable binding of at least a part of this N-terminal sequence to an unknown nuclear protein, RNA, or DNA. In this study, we showed, using an antibody against the anti-BH3 peptide of Bax, that the BH3 sequence was not further degraded or translocated into the nucleus. The BH3 sequence is polymerized with calmodulin at 20 μM xanthurenic acid (Figure 5) and colocalized in the mitochondrial membrane despite cell death, destruction of mitochondria and mitochondrial membrane polymerization (Mitotracker-red staining) (Figure 6C).

We consider that the BH3 sequence bound to calmodulin in the presence of xanthurenic acid is covalently inserted into the mitochondrial membrane, leading to constitutive activation of caspase-9 and caspase-3 and destruction of mitochondria [11,12].

## Discussion

Epidemiological studies show that small molecules are associated with disease development. Many programs of disease prevention indicate that cigarette smoking and air pollution should be avoided to prevent diseases [20-22]. However, the mechanism of the pathology induced by small molecules has not been reported. Many diseases are associated with aging, suggesting that a posttranscriptional modification, developed over the life span, may be an essential factor. Covalent attachment of small molecules to proteins changes the protein folding, and the proteins become stably modified by the small molecules.

The terms "misfolded" or "unfolded" proteins are currently used interchangeably for the stably modified proteins or transiently modified proteins, making communication difficult between researchers in the field. Misfolded proteins need a clearer definition to permit progress in the understanding of disease development by the posttranscriptional mechanism. We propose to define "misfolded proteins" as stably modified proteins and "unfolded proteins" as transiently or reversibly modified proteins, and these definitions were used throughout this paper. In the current study, misfolded proteins are covalently and irreversibly modified by a small molecule, leading to a stable interaction between the proteins. The stable interaction occurs usually between the regulatory sequences and leads to erroneous signaling resulting in cell pathology.

We observed that the covalent modification of the proteins by small molecules is the mechanism of formation of a new covalent network in pathological apoptosis. The primary modification of the proteins preferentially occurs in the presence of substances that have or generate quinone-like structures. The upstream modifications are followed by mitochondrial damage and the attachment of simple carbonyls or lipids to the proteins under oxidative stress.

These quinones lead to enzyme-free crosslinking with the basic amino acids [23], as observed in the presence of xanthurenic acid [9]. Many substances inducing oxidative stress, such as MPTP [24] or streptozotocin, lead to activation of indoleamine-2,3-dioxygenase (IDO) via oxidative stress and formation of kynurenines [25]. Then, a primary cell culture in the presence of xanthurenic acid is a good model to study the pathology associated with a posttranscriptional modification of proteins.

The increase in the incidence of metabolic diseases with aging is consistent with the observation in cell culture, in which the irreversible modifications of the proteins by small molecules are dependent on the time of incubation and the concentration of small molecules in the cell culture. The small molecule, xanthurenic acid, is formed from tryptophan during oxidative stress due to activation of IDO. IDO is increased in many degenerative disorders such as cancer, immunosuppressive diseases, and infectious diseases [26-29]. IDO overexpression is induced by emotional stress, IFN-γ, and oxidative stress [8,30,31]. However, IDO scavenges oxygen radicals, which are known to play a role in disease development [7,32]. Tryptophan degradation by IDO leads to production of nicotine amide or, alternatively, xanthurenic acid. Kynurenine aminotransferase (KAT) is the enzyme leading to the alternative pathway of xanthurenic acid formation. KAT also has allosteric enzymatic activity for glutaminase K and cystathionine-β-lyase [33,34]. The best inhibitor of KAT thus far is isonicotinic acid hydrazide, which is used for the treatment of tuberculosis [35]. Xanthurenic acid has been reported in pathological conditions such as infection [36], Crohn's disease [30], cataract [13], and depression and anxiety [37,38]. Previously, xanthurenic acid, the endogenous substance affecting protein folding, was reported to lead to pathological apoptosis [10]. HIV infection, causing AIDS, is an *in vivo *proof of the development of aging-associated disease due to infection and subsequent induction of IDO [39]. IFN-γ and ROS are widely proven stimulators of the inducible enzyme IDO [30]. Oxidative stress leads to lysosomal modification, which leads to lysosomal storage diseases [40,41]. Lysosomal modification is observed in astrocytes in the presence of xanthurenic acid [16]. Mitochondrial damage and oxidative stress are largely observed in aging-associated cardiovascular diseases. Xanthurenic acid induces pathological apoptosis through mitochondrial damage via irreversible Bax insertion into the mitochondrial membrane, as shown in this study and in previous reports [12]. An increase in xanthurenic acid was reported in cataract [42], in diabetes [43], and oxidative stress was observed in diseases of the central nervous system [44].

Our previous studies showed that xanthurenic acid leads to covalent modification of the proteins. Any substance able to react with the regulatory sequences, corresponding to the intrinsic disorder sequences, which are responsible for the protein-protein interactions, abolishes physiological regulation and leads to metabolic disorders. The substances can be provided by small-molecule drugs, environmental toxins, cigarette smoke etc. The mammalian body does not have enzymes that can remove such modifications, and the modified sequences accumulate during the life span. Small molecules, able to react covalently with at least 2 secondary groups of amino acids of the regulatory basic sequences, lead to their polymerization. The incidence of metabolic diseases increases with aging, which is consistent with the observation in cell culture that the irreversible modifications of the proteins by the small molecules are dependent on the time of incubation and the concentration of the small molecules in cell culture. The small molecule xanthurenic acid is the model molecule for the formation of the network of misfolded proteins, called misfoldome, in the cell culture model.

The current study showed that in the cell line transformed with SV40, Bad was not proapoptotic, but Bax/Bak was necessary for caspase-3 activation in the presence of xanthurenic acid. In the primary retinal cell culture (RPE), Bax polymerized with calmodulin in the presence of xanthurenic acid. Moreover, the BH3 sequence of Bax modified by xanthurenic acid was specifically translocated to the mitochondrial membrane in the degenerating cells. Such a protein polymerization leads to a nonregulated translocation of Bax and irreversible mitochondrial damage. However, the lack of Bax/Bak leads to another heavy pathology-cell detachment.

Xanthurenic acid in the cell cultures leads to the covalent interaction of Bax or MARCKS with calmodulin. However, this interaction was different in the WT cell line compared to the knockout cells.

## Conclusions

This study reports that pathological apoptosis is associated with a posttranscriptional modification of the regulatory sequences. New protein-protein interactions were observed-the covalent interactions of calmodulin with calmodulin-binding sites. In a primary cell culture or in a cell culture transformed by SV40, the new, incorrect covalent protein-protein interactions occur when the regulatory sequences are modified by xanthurenic acid. The covalent binding of calmodulin to the regulatory sequences is a characteristic of this new covalent and pathological network.

However, in the transgenic cell lines, which are used frequently to study disease development, the protein-protein interactions were completely different from those in WT cell lines. Moreover, the interactions are different between a cell line and a primary cell culture. These results show that it is impossible to study a disease that develops by posttranscriptional modification of the proteins, such as aging-associated diseases or infections, in a transgenic animal model because the protein-protein interactions in the transgenic cells are different from those in the WT cells. There have been many studies on protein-protein interactions in transgenic cells or animals, and the models are used for drug development. The present study shows that the protein-protein interactions in transgenic or immortalized cells are different from those in the wild- type organism.

The new interactions between the covalently modified proteins establish a covalent network of proteins, which lead to the metabolic disorder, called pathological apoptosis. The hallmark of this new protein network is the covalent binding of calmodulin to calmodulin-binding sites. Therefore, a condition leading to the nonphysiological covalent interactions of the proteins leads to tissue pathology, such as the nonregulated apoptosis observed in all degenerative diseases. The process of degeneration will begin earlier in the life span if there was increased exposure to small molecules, which accumulate due to oxidative stress associated with infection, emotional stress, air pollution, dietary intake of proteins from older animals and perhaps transgenic plants/animals, cigarette smoke, or drug use. The modifications can occur in any cell and consequently lead to organ degeneration.

We used the term "unfolded protein" for proteins with transiently changed folding in the maturation process or in a post-transcriptional reversible process such as phosphorylation, methylation etc. These proteins are completely degraded in the protein turnover process. However, the proteins that we termed "misfolded" are covalently and irreversibly modified by small molecule, such as xanthurenic acid, and alter physiological protein-protein interactions. These molecules cause the covalent protein-protein interactions. Such interactions are stable then are nonregulated, and are responsible for the cell pathology. The polymerized sequences are not removed from the cell, but lead to permanent signaling for cell apoptosis, resulting in the degeneration of the cell but not to degradation of the modified sequences, which form the protein aggregates ("plaques") observed in degenerative diseases.

We conclude that aging-associated diseases develop by posttranscriptional covalent modification of the regulatory sequences modified by small molecules. Xanthurenic acid in a primary cell culture is a model showing results corresponding with the situation *in vivo*. The xanthurenic acid in the primary cell culture model mimics the changes of the protein-protein interactions *in vivo*, which lead to disease.

## Competing insterests

H. Z. Malina patents
